# “We shall count it as a part of *kyogero*”: acceptability and considerations for scale up of single dose chlorhexidine for umbilical cord care in Central Uganda

**DOI:** 10.1186/s12884-018-2116-3

**Published:** 2018-12-04

**Authors:** David Mukunya, Marte E. S. Haaland, James K. Tumwine, Grace Ndeezi, Olive Namugga, Josephine Tumuhamye, Halvor Sommerfelt, Joseph Rujumba, Thorkild Tylleskar, Karen Marie Moland, Victoria Nankabirwa

**Affiliations:** 1 Centre for Intervention Science in Maternal and Child Health, Center for International Health, University of Bergen, Bergen, Norway; 2 CISMAC, Center for International Health, University of Bergen, Bergen, Norway; 3 Department of Pediatrics and Child Health, Makerere University, Kampala, Uganda; 4 CISMAC, Center for International Health, University of Bergen, Bergen, Norway; 5 Department of Epidemiology and Biostatistics, School of Public Health, Makerere University, Kampala, Uganda

**Keywords:** Newborn care, Health-intervention, Scale up, Acceptability, Implementation, Chlorhexidine, Newborn infections, Scalability

## Abstract

**Background:**

Cleansing the umbilical cord with chlorhexidine reduces neonatal morbidity and mortality, particularly in communities where newborn deaths and home births are common. As a result, the World Health Organization and national authorities are advocating the scale up of this intervention. In order for such a scale up to be effective, it has to be acceptable to the targeted population. With the overall aim to clarify conditions for scale-up, this study explored the acceptability of single dose chlorhexidine solution for umbilical cord care among health workers and infant care providers in the districts of Kampala and Mukono in Central Uganda.

**Methods:**

This was a qualitative study that involved mothers of neonates enrolled in a chlorhexidine trial, nurses implementing the trial, key community members and opinion leaders in childcare. We conducted 30 in depth interviews (IDIs) with mothers (18), health workers (8), traditional birth attendants (2), a father (1) and a grandmother (1) and 4 focus group discussions (FGDs), 3 with mothers and 1 with health workers. We used qualitative content analysis to analyze our findings and borrow upon Sekhon’s model when presenting our findings.

**Results:**

Cognitive and emotional responses to chlorhexidine use included ease of use, and a perception that chlorhexidine reduced smell and abdominal colic. We also found that wider social and cultural factors were important to chlorhexidine use. These included cultural value put on quick separation of the umbilical cord as well as the practice of bathing the baby in a herbal mixture called *kyogero*. We also found that older relatives were key decision makers in umbilical cord care for newborns, but were seldom present during health workers’ counseling of mothers about hygienic care of the cord.

**Conclusions:**

The application of chlorhexidine on the umbilical cord stump at birth was acceptable as an addition rather than a total replacement of traditional substances. The scale up of chlorhexidine should consider how to accommodate local beliefs and practices in a way that does not compromise the effect of the intervention; encouraging mothers to delay the bathing of babies in *kyogero* could be one way of doing this.

## Background

Reduction of neonatal mortality is one of the priority areas in the sustainable development agenda [1, 2]. To attain the sustainable development newborn target, Uganda has the challenge of reducing neonatal mortality from 27/1000 live births [3] to 12/1000 live births [1] by the year 2030. In order to achieve this target, key causes of neonatal death have to be addressed. Newborn infections account for about a quarter of all newborn deaths [4]. Infections also contribute significantly to deaths from other causes such as prematurity [5]. The umbilical cord stump, (hereinafter umbilical cord) is one of the major routes of infection in the neonatal period and a significant number of systemic infections are believed to progress from umbilical cord infections [6].

Proper hygienic care of the umbilical cord is recommended by the World Health Organization to reduce umbilical and systemic newborn infections [7]. Currently, the Ugandan Ministry of Health recommends dry umbilical cord care to discourage mothers from putting unclean substances on the umbilical cords, and this is in agreement with the World Health Organization (WHO) recommendations [7]. However, studies done in Uganda show low levels of adoption of the recommended dry umbilical cord care practice, with most authors quoting an uptake less than 50% [8–10]. Dry umbilical cord care seems to conflict with popular beliefs and cultural practices [11–13]. A common practice is to apply various substances on the umbilical cord to hasten umbilical cord separation [14].

Chlorhexidine, a topical antiseptic [15], is advocated for use in health facilities as an alternative to dry cord care in areas with high neonatal mortality and where dry cord care is unacceptable by the community [12]. Application of chlorhexidine to the umbilical cord has been found to reduce the incidence of both newborn infections and newborn deaths in Asian countries [6, 16–18] and is listed as a prioritized intervention in newborn health [5, 19]. A trial assessing the effect of a single application of chlorhexidine on the risk of infection of the umbilical cord stump (omphalitis) and newborn severe illness is currently underway in Uganda [20], and it is within this trial that we conducted this acceptability study.

Sekhon et al.*(2017)* have defined acceptability of healthcare interventions as a multifaceted construct that reflects the extent to which people delivering or receiving a healthcare intervention consider it appropriate, based on anticipated or experienced cognitive and emotional responses to the intervention [21]. The authors propose seven aspects to evaluate acceptability; individual’s affective attitude, burden, intervention coherence, ethicality, opportunity costs, perceived effectiveness and self-efficacy [21].

In this paper we draw upon Sekhon’s framework to explore the acceptability of single dose chlorhexidine solution for umbilical cord care among infant care providers and health workers in the districts of Kampala and Mukono in Central Uganda. With the overall aim to clarify conditions for scale-up we bring attention to social and cultural factors and argue their centrality for the emotional and cognitive responses addressed by *Sekhon* et al.

## Methods

This qualitative study was embedded within the chlorhexidine randomized controlled trial (RCT) designed to assess the efficacy of a one time application of chlorhexidine (4%) on the umbilical cord in reducing the incidence of neonatal severe illness [20]. The RCT is undertaken at three health centers in or close to Kampala, the capital of Uganda, and recruits newborns on the first day of life. After obtaining informed consent from the mother, research nurses/midwives apply the chlorhexidine solution on the umbilical stump of the newborns that are randomized to receive such care. Mothers are instructed not to put any substance onto the umbilical cord after the chlorhexidine has been applied, and only to wash it with plain water to remove dust and dirt if necessary. The RCT aims to recruit approximately 4700 newborns over a 3-year period. The newborns are followed up for 28 days and are examined on days 1, 3, 7, 14 and 28. The newborns in the control arm receive the standard of care, which is dry cord care with the same instruction not to apply anything on the umbilical cord, and only use plain water when bathing the baby. To the best of our knowledge, the RCT is the first project to introduce chlorhexidine for umbilical cord care in the study area and the participants did not have prior experiences with chlorhexidine.

The qualitative study was conducted between June 2016 and January 2017 in Kampala and Mukono district and had both a health facility component and a community component. The sites were chosen because they were the sites of the CHX randomized controlled trial. The Baganda, who are Bantu people, inhabit the central region of Uganda, and constitute the largest ethnic group in the study area as well as in the country at large [22]. Kampala district is mainly urban, whereas Mukono district is partly peri-urban but mainly rural. About 30% of people in Mukono district live 5 km or more from the nearest public health facility. A dominant role of husbands in accessing maternal and child health care services has been observed in this region [13]. Approximately 20% of women over 18 years in Mukono are illiterate [23] and there a high proportion of girls aged 12–19 years have given birth [23]. In Kampala, 98% are reported to attend skilled antenatal care with skilled, 94% deliver at a health facility, and 78% have a postnatal check within the first 2 days after birth [3, 23]. Traditional birth attendants (TBAs) are a popular source of knowledge about maternal and newborn care, but after the Ministry of Health terminated their previous working relationship with traditional birth attendants [24], their practice is considered illegal and liable to prosecution by local authorities [25]. Nevertheless TBAs remain popular and the communities protect them and continue to consult them for advice on pregnancy and newborn care [24]. As a result of their popularity, TBAs in the community where we conducted our study had been trained and incorporated into the village health team. Their main task was health promotion and referring women to the nearby government health center. The main groups of study participants are summarized in Table 1 below.

**Table 1:** Summary of Study participant’s in the chlorhexidine acceptability study in Central Uganda

	In-Depth Interviews (*N* = 22)	Focus Group Discussions (*N* = 4)
	Mothers-18	
	Healthworkers-8	Mothers - 3
	TBAs-2	Study nurses- 1
	Others-2	

Participants were chosen purposively, aiming for participants with rich experience in newborn care and for variation in experience and perspectives according to education, caretaker role and age. The health care workers recruited were study nurses who were employed specifically to work on the RCT. In addition, influential traditional birth attendants, a father and a grandmother were included. Two TBAs who also acted as members of the village health team, and therefore responsible for postnatal visits in their community, were given chlorhexidine bottles to use on babies in their communities and then interviewed. A father and a grandmother of a baby who had received chlorhexidine were also interviewed. This was done in order to enrich our data by soliciting views from various participants. All the participants had had an experience with chlorhexidine use for umbilical cord care. The TBA interviews were conducted in the community. We conducted 30 in depth interviews (IDIs) with mothers (18), health workers (8), TBAs (2), a father (1) and a grandmother (1) and 4 FGDs, 3 with mothers and 1 with health workers. We also video recorded the preparation of *kyogero*; a local herbal mixture composed of a variety of herbs, which are mainly in the form of leaves and stem barks. *Kyogero* is prepared by boiling these herbs to form a solution.

Two researchers; a social anthropologist and a medical doctor trained in qualitative research data collection; conducted the interviews. We used semi-structured interview guides for the different categories of study participants and topic guides for the different FGDs, which were both used flexibly and modified as need arose in the course of the study [26]. The interviews lasted between 20 to 80 min. We conducted most interviews in *Luganda*, the local language and a few in English mainly for health workers. A moderator and one note taker guided the focus group discussions (FGDs). We used video recording as a method of data collection to provide a detailed description and to increase the trustworthiness of the findings.

All the interviews were audiotaped and notes written down during the interview. The collected data was kept confidential and stored on a password-protected computer. A professional transcribed and translated interviews/FGD that were conducted in a language other than English. The principal investigator, who was present during all the interviews, proofread the transcripts comparing them to the audio recording. Data analysis was a continuous and iterative process guided by qualitative content analysis [27]. Two other independent researchers looked at the transcripts, categories and the subthemes generated. We used Nvivo 11.0.0 (QRS International, Cambridge, MA) to organize the analysis process. We borrowed upon Sekhon’s acceptability model when presenting our findings.

## Results

We present findings, which directly or indirectly may have influenced chlorhexidine use for umbilical cord care among our participants. We start by presenting the cognitive and emotional responses to Chlorhexidine use. Cognitive and emotional responses as a category are borrowed from Sekhon et al.*’*s framework for acceptability. In Table 2 we use constructs from the same framework when summarizing our findings. In the second part of the result section we present the social and cultural factors related to chlorhexidine use that underlie the cognitive and emotional responses.

**Table 2:** A summary of results from the chlorhexidine acceptability in central Uganda study, reported using constructs from the Theoretical Framework of Acceptability (TFA) by Sekhon et al

TFA construct	Definition	Finding (subtheme)	Anticipated impact on scale up and potential solutions
Affective attitude	Affective attitude implies how an individual feels about the intervention	Chlorhexidine is a pleasant solution	This will ease scale up
Burden and self-efficacy	Burden: The perceived amount of effort required to participate in the intervention Self-efficacy: Participant’s confidence that they can perform behavior required by intervention	Chlorhexidine is easy to use	This will ease scale up
Intervention Coherence and perceived effectiveness	Intervention coherence: Extent to which the participant understands the intervention and how it works Perceived effectiveness: Extent to which intervention is perceived to achieve it’s purpose	Chlorhexidine reduces bad smell Chlorhexidine prevents abdominal colic	This will ease scale up
Opportunity costs and Ethicality	Opportunity costs: Extent to which benefits, profits, or values must be given up to engage in the intervention Ethicality: Extent to which the intervention has good fit with an individual’s value system	‘Without *Kyogero* there is no blessing’: Desire to continue using *kyogero*	These are anticipated hindrances. A potential solution is to request participants to wait for umbilical cord separation before using *kyogero*.
		‘We shall call it value added kyogero’: Desire to add chlorhexidine to the potentially ‘unhygienic’ herbal solution	
		‘The cord should fall off quickly’: Desire for quick umbilical cord separation	This is an anticipated hindrance. A potential solution to this is to inform participants about the possibility of prolonged umbilical cord separation and emphasize that there is no danger with this.
		‘It is my mother who decides’: Multiple powerful actors and decision makers in the newborn period	This is an anticipated hindrance. A potential solution is involvement of elderly relatives in the intervention scale up.

### Cognitive and emotional responses to chlorhexidine use

#### ‘Chlorhexidine is easy to use’

Participants considered chlorhexidine use for the umbilical cord very convenient, particularly the availability, timing of application, preparation, and ease of application was highlighted. Chlorhexidine was readily available as it was found at the facility and distributed at the time the mother needed it. The substances otherwise used, ranging from plantain ash to complicated herbal formulations, were not as available and had to be brought and prepared by older caretakers like grandmothers and aunties. As one mother commented:

*Those that I tell about this drug see it as a good thing because some of them find it complicated to get transport fares to go and consult their grandparents (*Mother IDI).

Single dosage chlorhexidine was perceived as convenient, as caretakers did not have to remember timing of dosage. Health workers experienced chlorhexidine to be convenient compared to the salty water they had previously been using since it did not require boiling or other time-consuming preparation. Likewise a traditional birth attendant told us:

*It is very easy. You just open the bottle and then apply it. I think even the mothers can do it. The chlorhexidine is packed very well and there is no direct contact with fingers. Even if someone is not so clean, they can apply it (TBA IDI).*

In addition, the participants reported they liked the colour, smell and formulation of the chlorhexidine used in the study. They were very happy with the evaporating nature of the drug on the skin, leaving no trace of drug application.

#### It reduces bad smell

Participants reported that chlorhexidine was better at dealing with foul umbilical cord smell compared to alternatives. Mothers reported that the umbilical cord usually produced a foul smell (both when on the baby and off the baby), which they disliked because it gave an impression that the mother was unhygienic. The smell also hindered visitors from holding the baby. Some mothers also attributed the hesitancy of their husbands to hold the newborn to the foul smell from the umbilical cord. When the umbilical cord was off the baby, the smell attracted rats to the cord remnant and sometimes the rats took it. Anything that could prevent the umbilical cord from smelling was very welcome. Chlorhexidine seemed to do just that and was hence appreciated by most mothers. The umbilical cord smell could also predispose the child to harm and dangers as a grandmother told us:

*Now I realized is that this drug is better than our herbal medicine. With our herbal medicine the cord lasts for four days but the cord smells a lot. There is a friend of mine whose child was killed by the rat because it ate the cord. If you have rats and you are not clean, that foul smell attracts the rats and they can bite the umbilical cord (Grandmother IDI).*

In addition to the reduced smell, participants felt that the cord dried up faster and looked less slimy as one health worker noted:

*When you put it on the baby’s umbilical cord, you see the changes immediately (Health worker IDI).*

This quickened drying was beneficial as it encouraged some husbands to participate in newborn care early on as a mother told us:

*When I told him that the cord had dried up he was surprised, he asked whether he was not going to harm the baby and I told him that there was no problem, so he carried him because he was also seeing that it was already dry (Mothers FGD).*

#### ‘This child did not complain of abdominal colic’

Another perceived benefit to the use of chlorhexidine was the reduction in the occurrence of abdominal colic. Most mothers expressed a challenge in dealing with abdominal colic, which prompted them to apply diverse herbal formulations to the umbilical cord to prevent it. Abdominal colic was in fact a major reason for applying herbal formulations onto the umbilical cord both before and after umbilical cord separation. Most mothers were often disappointed, as most recommended medicines did not help. A number of mothers who had nursed a baby before noted that the baby on whom chlorhexidine was applied did not experience abdominal colic. This notion was expressed both in the individual interviews and in the focus group discussions, however they did not have any explanations for this observation. A mother told us:

*compared to the previous baby, this child did not complain of abdominal colic (Mother IDI).*

### Social and cultural factors related to chlorhexidine use

#### ‘The cord should fall off quickly’

Mothers of babies in this area expressed unease with the umbilical cord of newborns. They feared the umbilical cord and perceived it to be very vulnerable. Hence, most mothers desired the umbilical cord to separate as fast as possible. Contrary to their desire, mothers who used chlorhexidine commonly reported delayed umbilical cord separation, although they did not necessarily attribute the delay to chlorhexidine as explained by a traditional birth attendant:

*The baby’s mother told me that the cord might take long to fall off because the mother ate a lot of cow’s hooves (mulokoni) while pregnant. They say that when you eat a lot of cow’s hoof, the cord takes long to dry up. There is one pregnancy where I ate a lot of cow’s hooves and the cord took long to dry up (TBA IDI).*

Unlike the mothers, most study health workers were convinced that the delayed umbilical cord separation was caused by chlorhexidine:

*For most of the babies that we applied chlorhexidine, there is delay in umbilical cord fall off. On Friday I had a mother, she was calling me and she was wondering why her earlier baby’s cords usually take around 3–7 days but this one has taken two weeks (Health worker IDI).*

Some health workers further reported that having experienced quicker umbilical cord separation with other alternatives like saline water, they were unlikely to adopt chlorhexidine after the study:

*If I had a child I would use the normal saline, I wouldn’t use the chlorhexidine (Health worker IDI).*

As a result of delayed umbilical cord separation, some mothers (specifically those most influenced by mothers-in-law and other older women) reported reverting to herbal formulations.

*I was with my mother in-law when the doctor was telling us not to apply anything else, but when she was bathing him the following day, she said that let’s put something so that the cord could fall off quickly (FGD mothers).*

However, some mothers stated that had they been forewarned, they wouldn’t have been as alarmed as they were. Health workers also shared that view as one told us:

*I think with proper health education and sensitization the mother can be patient since it doesn’t take a whole month - she can be patient for two weeks (Health worker IDI).*

#### ‘Without *kyogero* there is no blessing’

A theme that came up in our interviews and FGDs was the practice of bathing the newborn in a herbal solution called *kyogero. Kyogero* is a mixture of several herbs, primarily used to bath the newborn, but also occasionally put in the mouth and on the umbilical cord. After the baby is bathed, the remaining *kyogero* is kept and re used for one to two weeks because it is very cumbersome and expensive to prepare. When applied on the umbilical cord, *kyogero* was believed to hasten umbilical cord separation:

*It is the kyogero that facilitated the breaking of the cord. I did not apply anything else because I hear people saying that other things are dangerous to the umbilical cord (Mothers IDI).*

Furthermore, *Kyogero* was perceived to immunize the child against common newborn diseases and to clear a common newborn skin rash. However, the purpose of *kyogero* went far beyond the prevention of physical illness. Even more importantly *kyogero* was composed of herbs (*ebombe* and *lweza*) that were believed to bring luck in the life of the newborn. As one TBA put it: *We use these [herbs] because they are the owners of blessings (TBA IDI).* Furthermore kyogero was believed to improve the intellect of a child, and make the child grow into a calm, obedient and respectful person.

There was also a perception that children who had not been bathed in *kyogero*, could turn out unruly and disrespectful:

*Those are the children you find in Kampilingisa (juvenile detention facilities) because they are stealing phones, bathing sponges, and other things. So you see the child is not stable (TBA IDI).*

It was also believed that the blessings of *kyogero* could manifest in several ways also later in life, not the least in getting a good marriage partner *‘for example a girl getting married to a rich man who also has good manners’ (TBA IDI).*

#### ‘We shall call it value added *kyogero*’

Participants were not willing to abandon *kyogero* because of its multiple benefits and instead often combined it with chlorhexidine:

*We still want our blessings, we want our peace, we want everything, we want the baby’s health and all this is in kyogero. We prevent many diseases through kyogero. So they [chlorhexidine and kyogero] will be able to work together. So it will be like chlorhexidine has added on to the benefits of kyogero. We shall count it as a part of kyogero. We shall call it value added kyogero (TBA IDI).*

Several mothers reported using substances on the cord alongside chlorhexidine, but they usually started this process after the umbilical cord had separated.

Although older participants insisted that the bathing in *kyogero* should commence on the first day, there was an appreciation of the delays in obtaining *kyogero* and hence using it later was also accepted. The younger mothers reported that they often initiated the bathing after the first week, usually when the umbilical cord had separated. When participants were questioned whether they could delay bathing the child in *kyogero* until the umbilical cord had separated, most of them replied affirmatively as a grandmother reported:

*We did not bath the baby with kyogero before the cord had fallen off because they (health workers) refused us (to do it). We bathed him with kyogero after the cord had fallen off. What the doctors have to tell the people is that they should wait for the cord to fall off; then start using kyogero on the babies because it is not so late by that time (Grandmother IDI).*

#### ‘It is my mother who decides’

Primarily grandmothers or other older female relatives known to have experience in newborn care usually made decisions concerning care of the newborn. This was specifically true for young mothers as one 19-year-old mother told us:

*Since it was my first born, my mother-in-law knew everything and she was the one who bathed him because we did not know how to do it. She would ask for some salt and cotton and you could not refuse her to apply it (Mother IDI).*

When asked about cord care, one young mother told us:

*I did not see it properly because it wasn’t me who was bathing the baby. At that time, I was afraid of him because he was so small. (Mother IDI).*

Some mothers reported that they first had to inform their husbands before committing to use chlorhexidine:

*When they explained to me, I told the doctor that I was not going to decide on my own. “Let me call the child’s father and if he accepts then it will be okay” (Mothers FGD).*

## Discussion

The findings suggest that chlorhexidine use for the umbilical cord is generally well accepted but that there are customary practices like ritual washing of the newborn and preferences like quick detachment of the umbilical cord, which may hinder exclusive use of chlorhexidine for umbilical cord care.

Our participants commented favorably on the physical attributes of chlorhexidine: color, smell and liquid formulation. This is in agreement with a study in Zanzibar, which showed preference in use of the liquid chlorhexidine formulation compared to the gel formulation [28]. Chlorhexidine use on the umbilical cord has been advocated as a means of discouraging unhygienic practices [12, 14, 29], especially in areas where dry cord care has been perceived as inappropriate [11, 12, 14]. In our study, we found that acceptance of chlorhexidine for umbilical cord care did not automatically lead to abandonment of the customary practices related to umbilical cord care. Participants were willing to abandon some of the substances applied directly onto the umbilical cord in favor of chlorhexidine, but were unwilling to abandon the washing of newborns in *kyogero* for the perceived benefits to the baby now and later in life*.* In effect, chlorhexidine was seen as an addition rather than a substitution to *kyogero* use for childcare. One of the reasons our participants were unwilling to abandon traditional substances applied to the umbilical cord, was their belief that these substances quickened umbilical cord separation.

One of the major barriers to chlorhexidine acceptance in our study was the suspicion among some participants and health workers that it delayed umbilical cord separation. Delayed umbilical cord separation following daily chlorhexidine for several days has been objectively assessed in a cluster randomized controlled trial conducted in Bangladesh, which revealed a 50% increase in umbilical cord separation time, when chlorhexidine was used [30]. A number of authors state that appropriate health communication may ameliorate the perceived negative effect of delayed umbilical cord separation [14, 30]. The perceived negative impact of prolonged umbilical cord separation may be rooted within deeper socio-cultural meanings attached to the cord, and may not solely be caused by a lack of adequate knowledge concerning chlorhexidine side effects. The findings of negative perceptions among study nurses and midwives who had previously been adequately trained in the chlorhexidine side effect gives credence to our assertion. Participants also reported that prolonged umbilical cord separation increased pressure from community members to resort to the herbal formulations previously used to speed up umbilical cord separation. This may obliterate the beneficial effects of chlorhexidine in reducing umbilical cord infections.

Sekhon et al. have stated that acceptability of health care interventions depends on the the appropriateness of the intervention in terms of anticipated or experienced cognitive and emotional responses [21]. They state 7 constructs, which include: affective attitude (how an individual feels about the intervention), burden (perceived effort required to participate in the intervention), ethicality (intervention’s goodness of fit with individual’s value system), intervention coherence (participant’s understanding of how intervention works), opportunity costs (benefits, profits and values to be given up to engage in the intervention), perceived effectiveness (perception that intervention has achieved purpose), and self efficacy (participant’s confidence that they can perform the intervention) [21]. Our participants generally felt good, clear, and unburdened by the application of chlorhexidine. They were also confident of the effectiveness of the intervention, and did not find any major value conflicts in using chlorhexidine. Interestingly, we found that mothers perceived chlorhexidine to be effective in prevention of umbilical colic, a finding that we cannot explain biologically. Possible hypothetical explanations include the reduction in umbilical cord colonization with microorganisms resulting in reduced colic, or a placebo effect resulting from chlorhexidine application to the umbilical cord. Despite mothers having serious opportunity cost challenges in having to forfeit herbal formulations, we argue that the intervention was considered largely acceptable on the individual level. However, this was not adequate to ensure use. We came across incidences where despite cognitive and emotional appropriateness of chlorhexidine as experienced by mothers, the product was not approved until accepted by significant others with decision making power in newborn care. These significant others were mainly older female relatives. Hence, widespread chlorhexidine acceptance may not be achieved unless these older relatives who are the practical caretakers and decision makers approve of it. In addition to Sekhon’s concept of acceptability as cognitive and emotional appropriateness, [23] we suggest to include social and cultural appropriateness as a condition for acceptability. Even if the social is seen as implicit in the concepts of affective attitude, opportunity costs, and self-efficacy, we argue that the social and cultural dimension is undervalued in Sekhon’s model and that it should be given more weight. This is particularly relevant in contexts where decision-making and implementation of a health care intervention is not in the hands of the target group - in this case, the mother.

We enlisted TBAs in the study because we regarded them as opinion leaders in the community. Indeed, it seemed the TBAs were very influential as regards umbilical cord care and they reported favorably on their chlorhexidine experience. On the contrary, health workers, who are another group of opinion leaders, were apprehensive concerning chlorhexidine scale up. They expressed concerns with the prolongation of the umbilical cord separation. The apprehension by health care workers could potentially affect the sustainability of the chlorhexidine scale up. We also found that older women (mother’s mothers and mothers in law and sometimes neighbors) whom the mothers were in daily contact with influenced newborn practices, including cord care, much more than the community opinion leaders such as TBAs. This finding is not new, and has been reported from other African settings [31]. It has been argued by Kumar and colleagues that interventions to change newborn care behaviors should be targeted to households, and not to individuals [32]. However, none of the health facilities we visited had newborn health promotional activities targeted to persons other than the mothers.

In this study we interviewed participants in a randomized controlled trial, who were exposed to more information on the issue at stake, and hence probably became more health attentive than others [33]. In addition, since this randomized controlled trial recruited participants who gave birth in a health centre, the applicability of our findings to mothers who give birth at home may be limited. We mainly focused on individual conditions that could favor chlorhexidine scale up and did not tackle larger aspects like economic factors (for example cost of the product) and political factors (for example umbilical cord care policies) These are some of the limitations of our study. To increase the trustworthiness of our findings, we triangulated the data collection methods by using IDIs, FGDs and video, which broadened our understanding of perspectives about chlorhexidine acceptability [34].

## Conclusion

Our participants generally accepted umbilical cord care with chlorhexidine cleansing due to its convenience, and superiority in the reduction of umbilical cord foul smell and perceived abdominal colic facilitates scale-up. However, the perceived prolongation of umbilical cord separation associated with chlorhexidine was a discouraging factor, and could affect the sustainability of possible scale up efforts in the future. We recommend prior communication about its possible effect of delayed umbilical cord separation. Despite willingness to abandon some of the substances used for umbilical cord care, participant were unwilling to abandon the practice of bathing the newborn in the local herbal solution *kyogero,* in order to use chlorhexidine exclusively. However, they were willing to combine chlorhexidine with *kyogero* to harness the more holistic benefits of *kyogero,* which include future wellness and blessings. Participants’ willingness to defer the bathing of newborns in *kyogero* to a period after umbilical cord separation, offers an opportunity for chlorhexidine scale up efforts. Special attention should be paid to key decision makers like grandmothers and mothers in-law, who are often ignored in the current childcare promotion activities, but were found to be very important in the care of newborns, especially during the first few days after birth. According to our study participants, it is the people closest to the mother of the newborn who seem to be more influential in terms of cord care decision-making than the TBAs and the health workers that we originally had identified as opinion leaders. The identification of a significant bathing practice in a study designed for umbilical cord care, highlights the importance of an explorative approach to implementation science, as the factors that influence adoption of an intervention may be beyond the boundaries of what we initially set out to explore.

## Abbreviations

CHX: Chlorhexidine
FGD: Focus Group Discussion
IDI: In Depth Interview
RCT: Randomized Controlled Trial
TBA: Traditional Birth Attendant
WHO: World Health Organization
